# Durable LVAD therapy in end-stage methamphetamine-associated heart failure: a case report of the bridge to opportunity and transplant candidacy

**DOI:** 10.1093/ehjcr/ytag498

**Published:** 2026-07-13

**Authors:** Cooper B Kersey, Jennifer L Azen, Lisa Wiederspan, Sunny Rockom, Jennifer A Beckman, Salpy V Pamboukian, Sunu S Thomas

**Affiliations:** Division of Cardiology, Department of Medicine, University of Washington School of Medicine, 1959 NE Pacific Street, Box 356422, Seattle, WA 98195-6422, USA; Division of General Internal Medicine, Department of Medicine, University of Washington School of Medicine, 325 Ninth Ave, Box 359780, Seattle, WA 98104, USA; Division of Cardiology, Department of Medicine, University of Washington School of Medicine, 1959 NE Pacific Street, Box 356422, Seattle, WA 98195-6422, USA; Division of Cardiology, Department of Medicine, University of Washington School of Medicine, 1959 NE Pacific Street, Box 356422, Seattle, WA 98195-6422, USA; Division of Cardiology, Department of Medicine, University of Washington School of Medicine, 1959 NE Pacific Street, Box 356422, Seattle, WA 98195-6422, USA; Division of Cardiology, Department of Medicine, University of Washington School of Medicine, 1959 NE Pacific Street, Box 356422, Seattle, WA 98195-6422, USA; Division of Cardiology, Department of Medicine, University of Washington School of Medicine, 1959 NE Pacific Street, Box 356422, Seattle, WA 98195-6422, USA

**Keywords:** Addiction medicine, Methamphetamine-associated heart failure, Left ventricular assist device, Heart failure, Case report

## Abstract

**Background:**

Methamphetamine-associated heart failure (MAHF) is an increasingly recognized cause of severe non-ischaemic cardiomyopathy with a 231% rise in hospital admissions from 2008 to 2020. MAHF is associated with high morbidity and mortality, and advanced therapy options are challenged by substance use and addiction.

**Case summary:**

A 40-year-old male with advanced MAHF and active substance use presented with out-of-hospital cardiac arrest requiring escalating temporary mechanical circulatory support and ultimately durable left ventricular assist device (LVAD) placement as a bridge to candidacy strategy. After 6 months of documented abstinence from substances and engagement in outpatient addiction medicine treatment, his Stanford Integrated Psychosocial Assessment for Transplant (SIPAT) score had improved from 53 to 23, reclassifying him from a ‘poor’ to ‘minimally acceptable’ transplant candidate. He underwent successful cardiac transplantation and continues to be abstinent at 21 months post-transplantation.

**Discussion:**

This case demonstrates that active substance use represents a modifiable rather than absolute barrier to cardiac transplantation when addressed through a structured multidisciplinary framework, the bridge to opportunity. In the bridge to opportunity, durable LVAD implantation allowed for the haemodynamic support for a patient with end-stage MAHF to engage in outpatient addiction medicine care and demonstrate sustained sobriety. Ethical concerns regarding equitable organ allocation are best addressed through individualized psychosocial risk stratification using validated tools such as the SIPAT, rather than categorical exclusion of patients with substance use disorders. The bridge to opportunity is a reproducible model for appropriately resourced multidisciplinary transplant programmes and appropriately selected patients.

Learning points

- Substance use is a modifiable barrier to transplantation with appropriate addiction medicine care.
- Durable left ventricular assist device (LVAD) therapy can provide effective haemodynamic support in select patients with advanced methamphetamine-associated heart failure.
- A multidisciplinary approach to patients with heart failure and substance use allows for a clinical pathway for LVAD as a bridge to sobriety and cardiac transplantation.

## Primary specialities involved other than cardiology

Addiction medicine and cardiac surgery.

## Introduction

Methamphetamine-associated heart failure (MAHF) is an increasingly recognized cause of severe cardiomyopathy and is associated with substantial morbidity and mortality.^1^ Chronic methamphetamine use exerts direct cardiotoxic effects through catecholamine excess, oxidative stress, and microvascular dysfunction, leading to progressive myocardial remodelling and impaired systolic function.^2^ Patients with MAHF often present at a younger age with advanced heart failure symptoms, recurrent hospitalizations, and limited physiologic reserve.^2^ Management of advanced MAHF is particularly complex, as consideration of guideline-directed medical therapy (GDMT), mechanical circulatory support (MCS), or cardiac transplantation is complicated by ongoing substance use, psychosocial instability, and concerns regarding adherence. These challenges underscore the need for multidisciplinary care approaches and highlight important clinical and ethical considerations when evaluating advanced heart failure therapy options in this high-risk population.

## Summary figure

**Figure:**
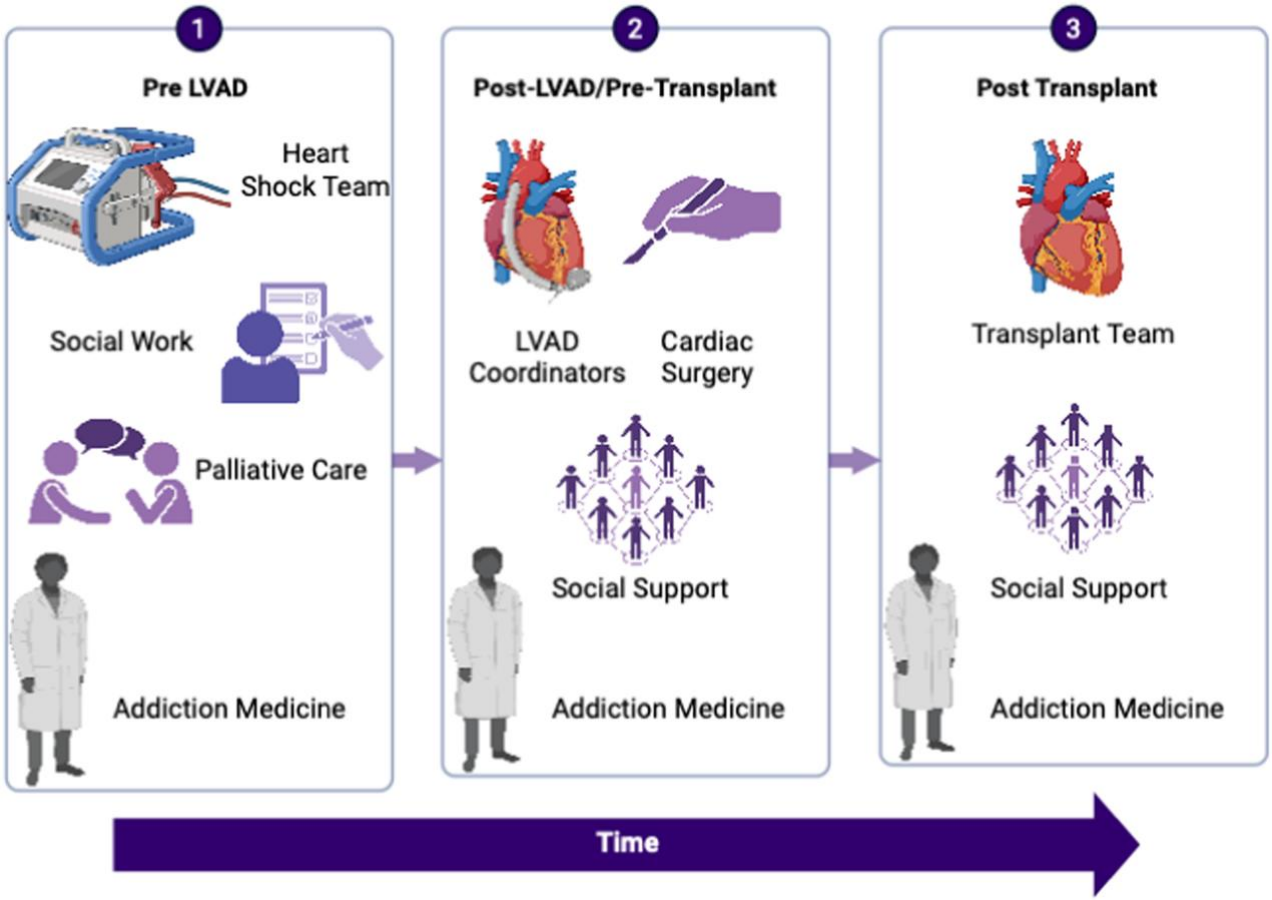
Clinical pathway for advanced methamphetamine-associated heart failure (MAHF) using a bridge to opportunity model. The model includes durable LVAD implantation, longitudinal addiction care, and psychosocial vetting leading to transplant candidacy.

## Case presentation

A 40-year-old male with a history of substance use (methamphetamine, alcohol, tobacco, and Adderall) and a non-ischaemic cardiomyopathy (left ventricular ejection fraction (LVEF) 20%) due to MAHF on home inotropes presented with an out-of-hospital cardiac arrest due to ventricular fibrillation. He was placed on venoarterial extracorporeal membrane oxygenation for MCS due to electrical storm. Anti-arrhythmic therapy with lidocaine and amiodarone led to electrical quiescence and the patient was transitioned to a surgically implanted Impella 5.5 for left ventricular support. The patient was unable to be weaned from MCS over the coming weeks and a multidisciplinary conference between heart failure, cardiac surgery, addiction medicine, and social work was held to discuss the most appropriate long-term therapy for his Stage D heart failure. Active substance use presented an acute barrier to cardiac transplantation, and a durable left ventricular assist device (LVAD) as a bridge to candidacy strategy was pursued based on a favourable recidivism risk assessment by addiction medicine. His post-LVAD implant care plan required fulfilment of a substance use agreement that consisted of 6 months of documented abstinence from all substances on biweekly urine toxicology screens and monthly phosphatidyl ethanol blood tests.

The patient had been diagnosed with heart failure 2 months prior to his cardiac arrest. He was unable to tolerate GDMT and suffered multiple heart failure exacerbations, ultimately leading to the initiation of home inotrope. He underwent a coronary angiogram and serologic work-up, which did not reveal a cause for his heart failure. Given his significant substance use history, his cardiomyopathy was attributed to stimulant use. In terms of his history of substance use, he socially used alcohol, tobacco, and stimulants during his adulthood. Two years prior to his heart failure diagnosis, he suffered a family tragedy, and his substance use escalated to heavy alcohol use (up to 17 standard drinks in a day) with regular methamphetamine use. He also used cannabis, tobacco, and opiates intermittently.

The post-LVAD psychosocial assessment by addiction medicine identified a favourable recidivism risk profile based on a short duration of heavy substance use (less than 5 years), high patient motivation, and strong social support. Post-discharge, the patient engaged in intensive outpatient counselling with an addiction medicine specialist every 2 to 3 months in tandem with structured toxicology monitoring. Consistent attendance and complete abstinence across all monitoring intervals were prerequisites for transplant candidacy. Six months of biweekly urine toxicology screens and a monthly phosphatidyl ethanol level corroborated his complete abstinence from all substances. An echocardiogram performed 6 months after LVAD implantation revealed a severely dilated left ventricle (LVIDd 7.4 cm) with severely reduced function (LVEF 11%) despite appropriate LVAD unloading (partial aortic valve opening with each cardiac cycle) (see Supplementary material online, *Image S1A* and *S1B*). Right ventricular function was mildly reduced by transthoracic echocardiogram with a fractional area change of 31%. An outpatient right heart catheterization showed persistent low-output heart failure and left-sided congestion with a right atrial pressure of 8 mmHg, pulmonary artery pressure of 35/18/25 mmHg, pulmonary capillary wedge pressure of 19 mmHg with V waves to 28 mmHg, and Fick-derived cardiac index of 2.0 L/min/m^2^ (see Supplementary material online, *Image S2*). Unfortunately, the patient suffered two methicillin sensitive staph aureus driveline infections requiring suppressive antibiotics. A third MSSA driveline infection while on suppressive antibiotics prompted the multidisciplinary advanced heart failure committee to discuss cardiac transplant as an option.

At the time of his third driveline infection, the patient had demonstrated 6 months of abstinence, broadening the possible therapeutic pathway to include three options: bridge to recovery, long-term LVAD therapy, or bridge to transplant candidacy. Based on the contemporaneous echocardiographic and haemodynamic data demonstrating low cardiac output and left-sided congestion despite appropriate unloading, myocardial recovery was unlikely. His history of recurrent driveline infections despite appropriate suppressive antibiotics made long-term LVAD therapy challenging. As a result, bridge to transplant candidacy was the preferred route for him. His addiction medicine assessment indicated a favourable recidivism risk based on 6 months of documented sobriety, and his Stanford Integrated Psychosocial Assessment for Transplantation (SIPAT) score of 23 classified him as a ‘minimally acceptable candidate’. He was ultimately listed for transplant contingent upon continuing to engage with addiction medicine and undergo routine urine toxicology screening.

The patient was successfully transplanted and at 21 months post-transplant he remained abstinent from all substances. The pathology of his explanted heart demonstrated interstitial fibrosis of both the right and left ventricle consistent with a non-ischaemic cardiomyopathy (see Supplementary material online, *Image S3*). Post-transplant the patient continued to engage with addiction medicine in the outpatient setting and undergo routine screening every 3 months with phosphatidyl ethanol levels and urine drug screens, all of which have resulted negative to date. Data sharing is not applicable to this article as no datasets were generated or analysed during the current study.

## Discussion

This case highlights the successful cardiac transplantation of a patient with MAHF after structured addiction medicine intervention while utilizing durable LVAD therapy for haemodynamic support. This case adds to the growing body of literature of transplant for complex patients and ethical allocation of donor organs. Multidisciplinary care models such as the proposed bridge to opportunity are needed as we see an increase in prevalence in MAHF and more cases of advanced disease. The prevalence of methamphetamine-associated heart failure has increased exponentially in recent years, with data from the national inpatient sample showing a 231% increase in MAHF hospital admissions from 2008 to 2020.^1^ The natural history of MAHF depends upon the duration of stimulant use and adherence to medical therapy, with repetitive exposure leading to left ventricular dilation, systolic dysfunction, and ultimately myocardial fibrosis.^2^ In its most severe form, patients with MAHF can present with cardiogenic shock and electrical instability requiring MCS. Based on the paucity of literature on cardiac transplantation for patients with substance use disorders in remission, this is an under-reported clinical pathway that needs to be highlighted.

Historically, patients suffering from addiction are not immediate candidates for advanced therapies such as LVAD and cardiac transplantation due to data demonstrating worse long-term outcomes in patients with substance use disorders.^3,4^ For this reason, data on the utilization of LVAD and transplant in patients with MAHF are limited—a case series describes two patients undergoing LVAD as a successful bridge to recovery strategy.^5^ Another study of patients with toxin-mediated cardiomyopathies identified four patients with MAHF who underwent LVAD implantation.^6^ The authors identified four factors associated with post-LVAD adherence to medical therapy and freedom from returning to methamphetamine use: acceptance of counselling and psychosocial support, addiction of <5 years, employment, and not selling drugs for financial support. These studies highlight that methamphetamine use disorder is akin to other medical contraindications to transplant (such as elevated body mass index or uncontrolled diabetes) with a gradation of risk that can be objectively quantified and modified with appropriate evidence-based interventions.

The transplantation of patients with substance use disorders raises important ethical considerations around the equitable allocation of scarce donor organs. A central concern is whether patients with addiction-related end-organ disease (alcoholic hepatitis and MAHF for example) will have worse post-transplant outcomes due to recidivism. The liver transplantation literature has grappled with this tension extensively; the historically applied 6-month sobriety rule for alcohol-associated liver disease has been challenged on the grounds that abstinence duration alone is a poor predictor of post-transplant return to use. In the case of acute alcohol-associated hepatitis, withholding transplantation based on the 6-month rule may result in preventable death in otherwise suitable transplant candidates.^7^ A meta-analysis of the liver transplant literature demonstrates that while recidivism after transplantation does occur, rates vary substantially based on pre-transplant psychosocial factors, underscoring the importance of individualized risk stratification over categorical exclusion of patients with substance use disorders.^8^ These data support a framework in which addiction is treated as a chronic, modifiable medical condition, and transplant candidacy decisions are guided by objective, validated psychosocial assessment tools, such as the SIPAT. The SIPAT score incorporates a patient’s readiness for transplant, social support team, psychological stability, and lifestyle/substance use in order to generate a score that classifies the individual’s candidacy on a spectrum from ‘good’ to ‘high risk’.^9^ In the case presented, the patient’s SIPAT score went from 53 at the time of his initial cardiac arrest to 23 after his 6 months of intensive addiction medicine care, reclassifying him from ‘poor candidate’ to ‘minimally acceptable’.

We propose that while active substance use is an acute barrier to cardiac transplantation because it predicts poor health-related quality of life and outcomes post-transplant, chronic addiction is a modifiable barrier with appropriate treatment.^10^ This statement is supported by the most recent ISHLT guidelines for the care of heart transplant recipients, which identifies chronic substance use as a modifiable psychosocial factor that can be addressed with the appropriate interventions.^11^ Substance use disorders occur along a spectrum of disease, and based on a clinical assessment, an expert in addiction medicine can develop a patient-specific treatment plan that takes the form of a substance use agreement and outpatient monitoring. This case demonstrates that substance use is a modifiable barrier to transplant with patient-specific risks of returning to use. A multidisciplinary approach with addiction medicine is imperative to identify patients who are appropriate bridge to opportunity and transplant candidates.

Active substance use is not considered an acute contraindication to either temporary MCS or durable LVAD, but a comprehensive psychosocial assessment prior to LVAD implantation to assess an individual’s ability to care for their device, manage medications, and make it to frequent medical appointments is crucial.^12^ In the case presented, the patient had a low risk of recidivism due to his motivation to engage in treatment of his addiction, strong psychosocial support, and relatively short duration of addiction. These protective factors made the patient an optimal bridge to opportunity candidate. For this patient, a durable LVAD provided the haemodynamic support necessary to get the patient to hospital discharge, where he could then have the time to demonstrate abstinence and engage in addiction medicine treatment.

**Figure 1:**
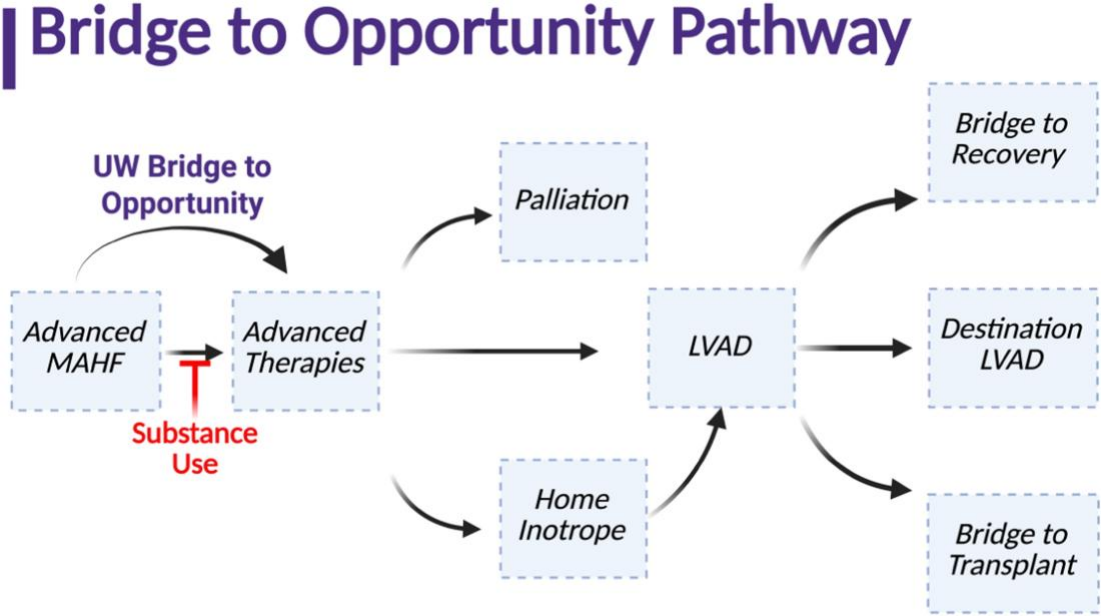
The bridge to opportunity model includes the full spectrum of advanced therapies and can be individualized to a specific patient’s clinical needs, psychosocial support, and goals of care.

The architecture of the bridge to opportunity pathway is complex and depends upon a multidisciplinary team that changes based on what section of the bridge the patient is traversing (*Central Figure*). In this case, time through LVAD therapy was the foundation for a bridge to opportunity, allowing for meaningful addiction treatment and demonstration of sustained commitment. The multidisciplinary care team that guides a patient across the bridge to opportunity is the scaffolding that rises from the strong foundation of time. Addiction medicine is a constant throughout this journey as they provide longitudinal care for the chronic disease of addiction and assess the patient’s risk of recidivism. The primary cardiology team will change depending upon what phase of the bridge patient is in: MCS team pre-LVAD, LVAD team including LVAD coordinators post-LVAD, and transplant team after transplant. Palliative care is essential to establish what a patient’s wishes and goals are early in this process because not every patient values longevity. No patient can cross this bridge alone and the social work assessment is essential to shedding light on what psychosocial support an individual has to accompany them as they cross the bridge to opportunity. The clinical pathway for advanced or end-stage MAHF must be tailored to both the individual patient and the resources of the treating health system. Furthermore, the bridge to opportunity model is a reproducible and scalable model for the care of patients with end-stage heart failure and addiction with the appropriate expertise and clinical resources (*Figure 1*). Each transplantation programme should evaluate their own institutional resources and approach to high-risk transplant patients in order to identify whether the multidisciplinary care for patients with MAHF is concordant with the resources and culture of the programme. Based on this case report and the available literature, the authors propose that the bridge to opportunity framework can be applied to select patients with MAHF in appropriately resourced multidisciplinary transplant groups. Future studies are needed to validate the bridge to opportunity model in larger cohorts and to define patient selection criteria that maximize outcomes across systems of care.

## Conclusion

This novel case of end-stage MAHF treated with durable LVAD as a bridge to transplant candidacy demonstrates that addiction can be addressed as a modifiable barrier to transplantation through a multidisciplinary care model, the bridge to opportunity. Addiction requires longitudinal care akin to any other chronic medical condition that potentially challenges a positive prognosis in a patient who requires advanced therapies. This multidisciplinary care is an active process of healing and preservation of sobriety that spans the pre-LVAD, post-LVAD but pre-transplant, and post-transplant phases of a patient’s journey across the bridge to opportunity.

## Supplementary Material

ytag498_Supplementary_Data

## Data Availability

The data underlying this article will be shared on reasonable request to the corresponding author.

## Lead author biography

Cooper B. Kersey, MD is an Assistant Professor of Medicine in the Division of Cardiology at the University of Washington School of Medicine. Dr Kersey’s research focuses on the intersection of cardiology and addiction medicine, with a particular emphasis on methamphetamine-associated heart failure. He utilizes qualitative research and implementation science to identify and address barriers to care in this high-risk population.

## Notes

**Consent:** Written consent was obtained from the patient for an anonymized case report using the EHJ Patient Consent Form and is being held by the treating institution.
