# Supplementary material for: Durable LVAD therapy in end-stage methamphetamine-associated heart failure: a case report of the bridge to opportunity and transplant candidacy
Source: Eur Heart J Case Rep. 2026 Jul 13;10(7):ytag498. doi: 10.1093/ehjcr/ytag498 (PMC13457941; doi:10.1093/ehjcr/ytag498)
Supplement: Supplementary file 1 [file ytag498_Supplementary_Data.pdf]

Supplemental Figures:

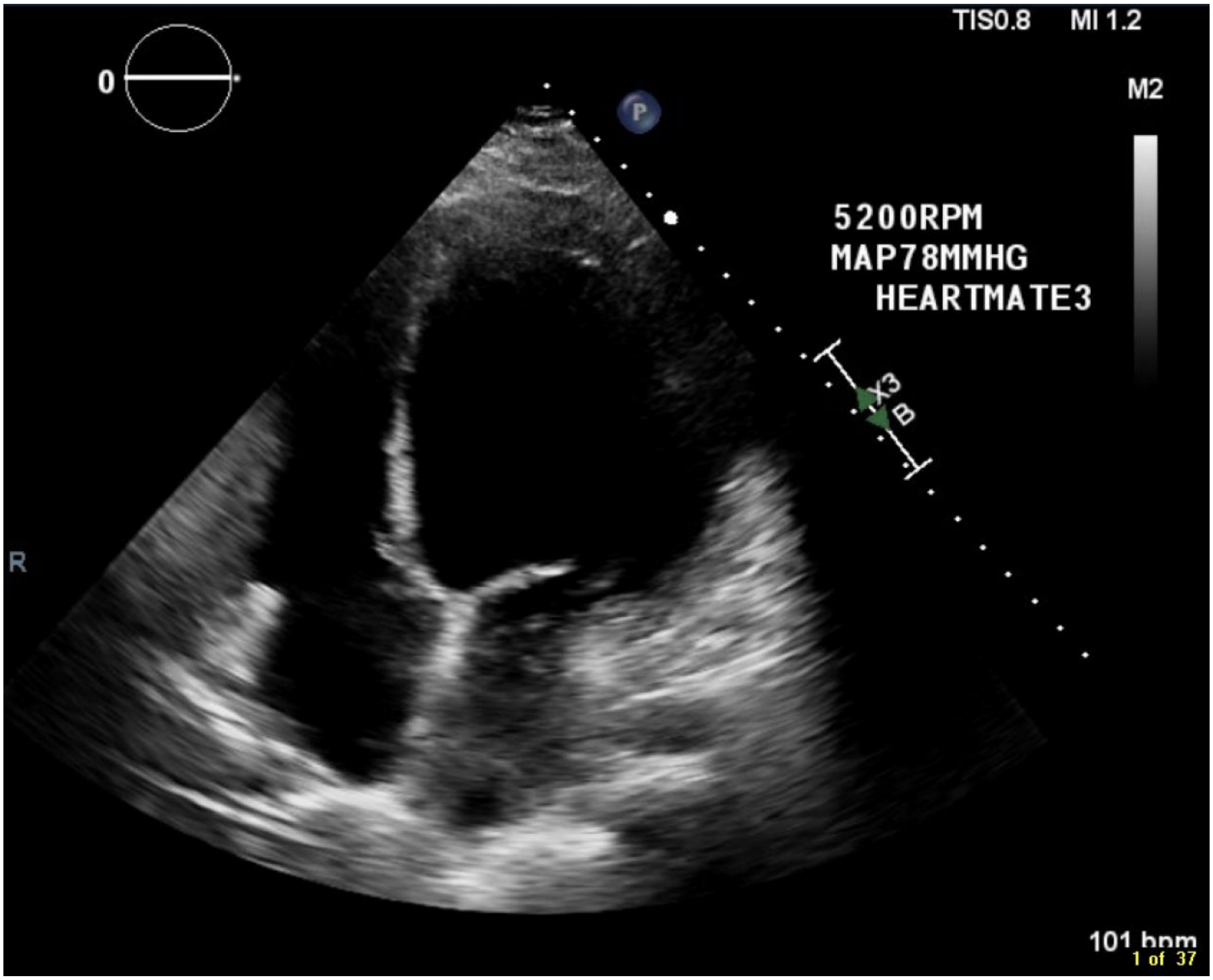

Supplemental Image 1a: Transthoracic echocardiogram performed after 6 months of left ventricular unloading with LVAD and abstinence from all substances. Apical four chamber is notable for a severely dilated left ventricle (LVIDd 7.4cm) and severely reduced systolic function (LVEF 11%)

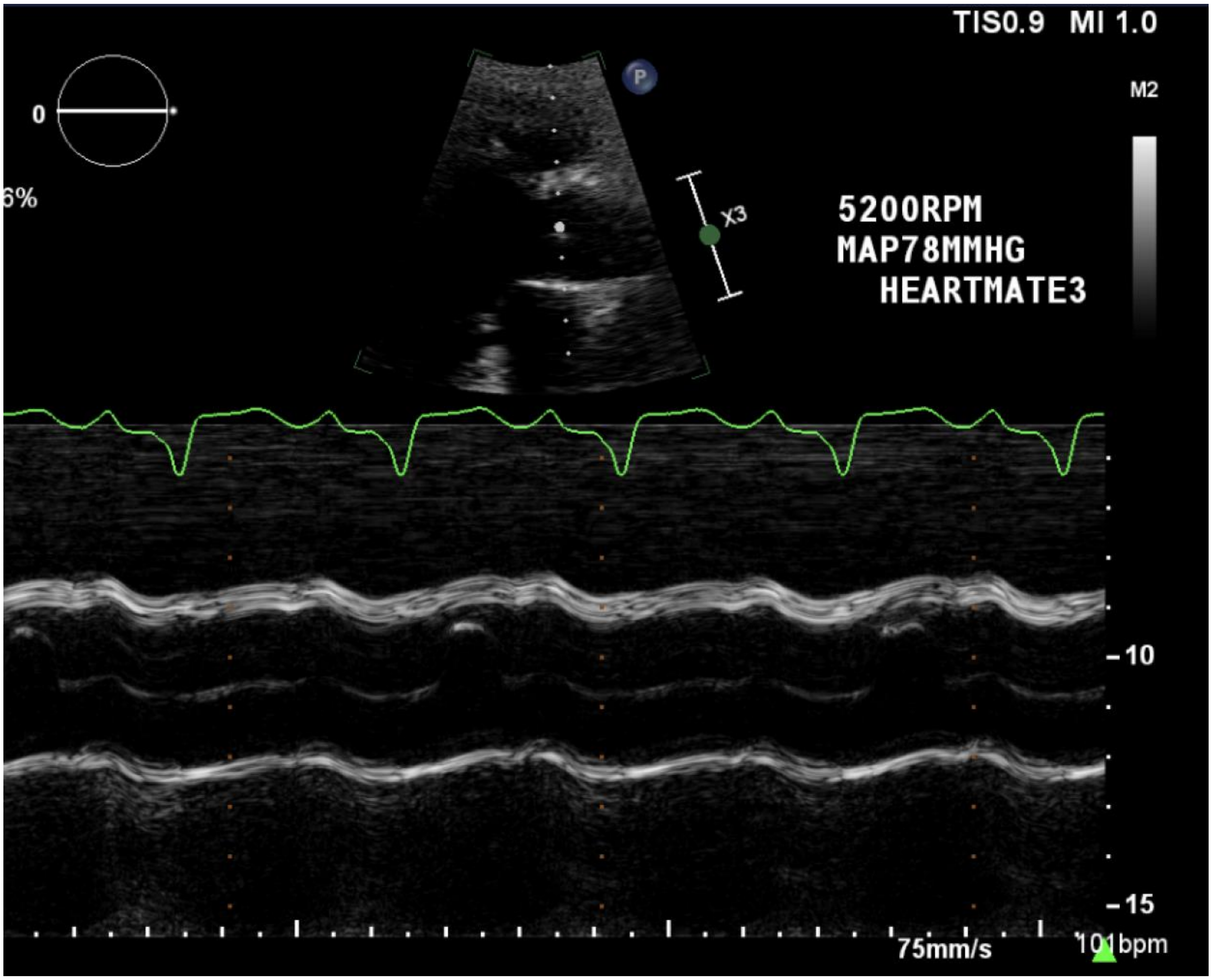

Supplemental Image 1b: Transthoracic echocardiogram performed after 6 months of left ventricular unloading with LVAD and abstinence from all substances. M-mode through the aortic valve demonstrating partial opening of the valve with every cardiac cycle.

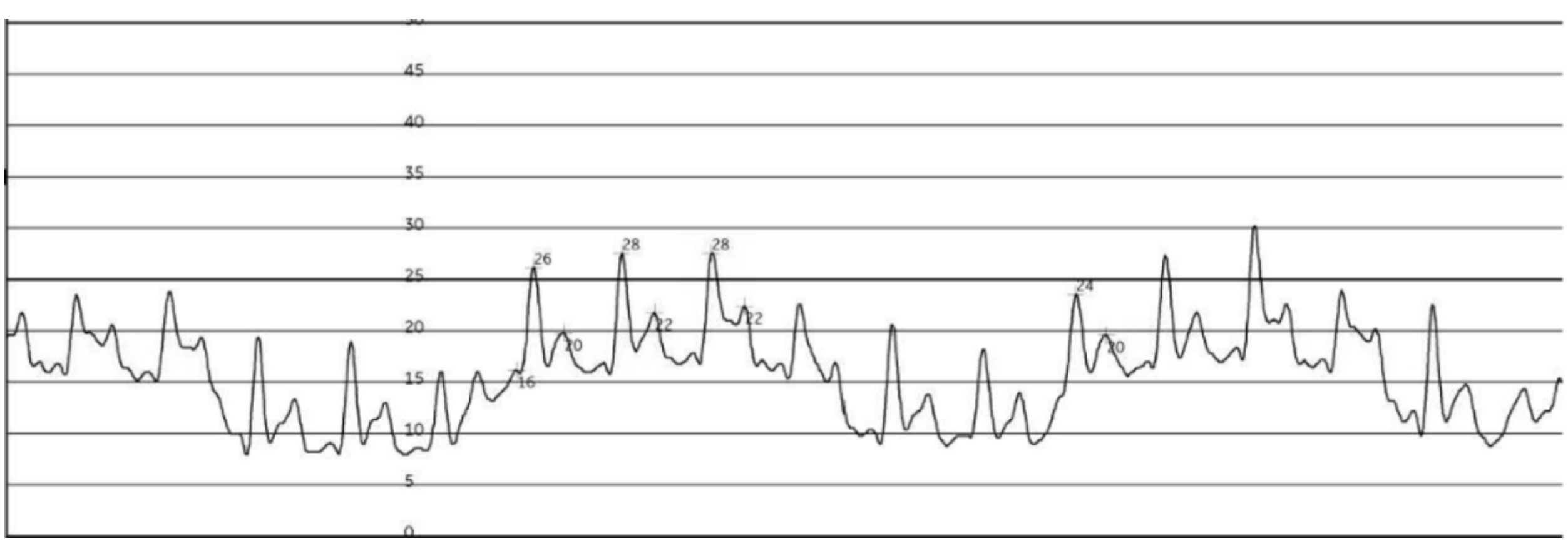

Supplemental Image 2: Right heart catheterization with LVAD at 5200rpm demonstrates a pulmonary capillary wedge pressure of 19mmHg with V-waves to 28mmHg.

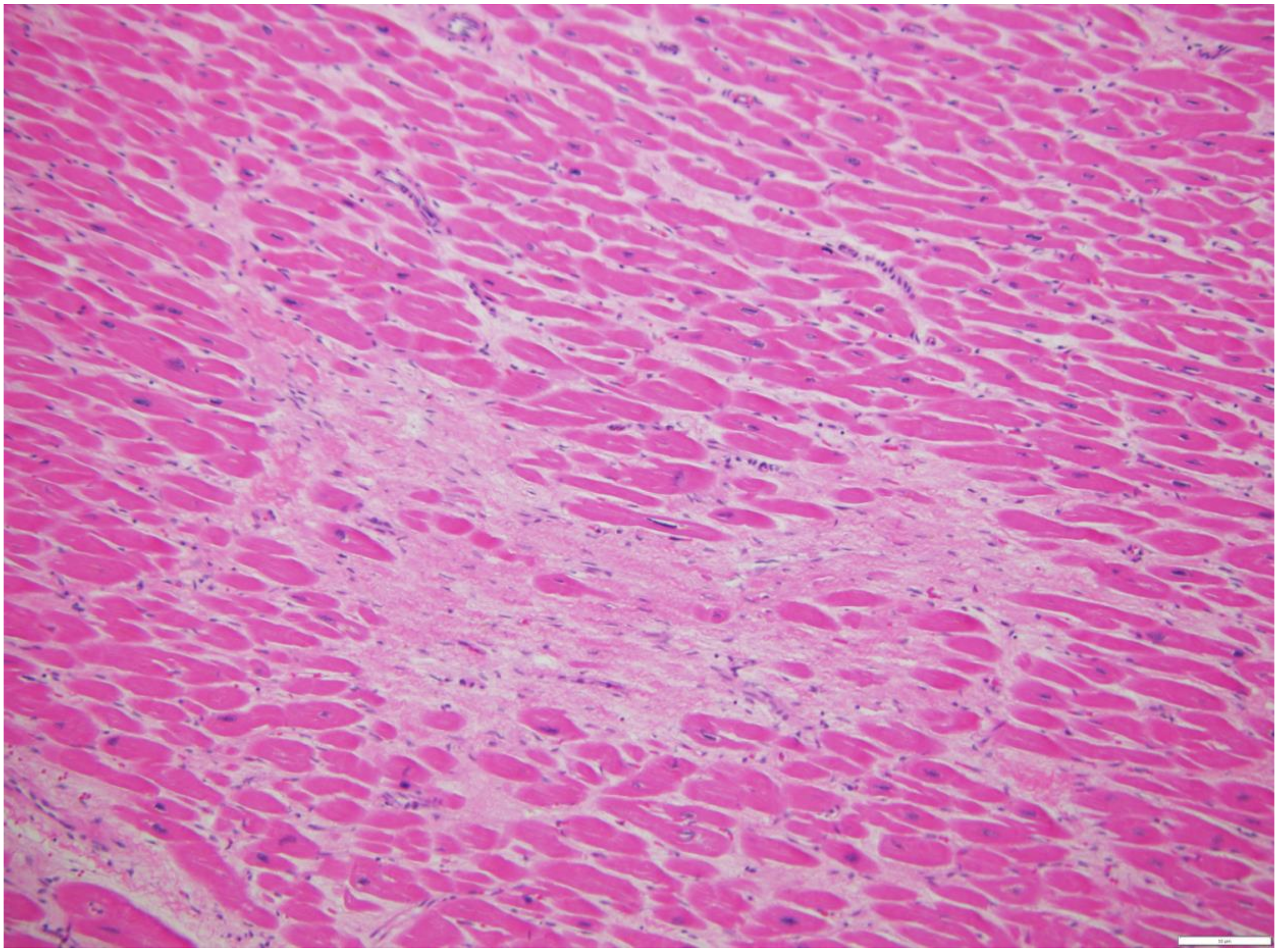

Supplemental Image 3: Pathology of explanted heart demonstrates interstitial fibrosis consistent with known non-ischemic cardiomyopathy.
